# The robotic PIANO: a critical narrative review of surgeon-controlled operative polyphony, human factors, and robotic training

**DOI:** 10.1007/s11701-026-04035-8

**Published:** 2026-10-07

**Authors:** Luigi Marano, Mirko Barone, Mehmet Mahir Özmen, Bojan Jovanovic, Francesco Paolo Prete, Silvia Malerba, Edmundo Inga-Zapata, Panagiotis Lainas, Elena Ruiz-Úcar, Miguel Angel Cáceres Yap, Vikas Jain, Beniamino Pascotto, Juan Santiago Azagra, Salaam Sadi, Ruslan Duka, Robert Molchanov, Adolfo Perez-Bonet, Nicolas Zucchini, Luis Osvaldo Suárez Carreón, Jaroslaw Skokowski, Adébusola Adenike Owokole, Archil Aladashvili, Miljana Vladimirov, Mahmoud Al-Balas, Cesar Santiago, Sharona Ross, Sharique Nazir, Yogesh Vashist, Adel Abou-Mrad, Mario Testini, Ludovico Docimo, David Albala, Rodolfo J. Oviedo

**Affiliations:** 1 Department of Medicine, Academy of Applied Medical and Social Sciences AMiSNS, Akademia Medycznych I Spolecznych Nauk Stosowanych) Ul. Lotnicza 3, 82-300 Elbląg, Poland; 2 Department of Surgery, Dnipro State Medical University, Volodymyra Vernadskoho St. 9, 49044 Dnipro, Ukraine; 3 Department of General Surgery, Anesthesia and Urology, Faculty of Medicine, The Hashemite University, Zarqa, Jordan; 4 Department of General Surgery and Surgical Oncology, “Saint Wojciech” Hospital, “Nicolaus Copernicus” Health Center, Gdańsk, Poland; 5 Department of General and Thoracic Surgery, SS. Annunziata University Hospital, Chieti, Italy; 6 Department of Medical, Oral, and Biotechnological Sciences, G. d’Annunzio University of Chieti, Chieti, Italy; 7 MOC Obesity & Cancer Center, Ankara, Turkey; 8 Hacettepe University Medical School, Ankara, Turkey; 9 Liv Hospital, Ankara, Turkey; 10 Center for minimally invasive surgery, University Clinical Center Niš, Niš, Serbia; 11 Department of Precision and Regenerative Medicine and Ionian Area (DiMePRe-J), School of Medicine, University of Bari Aldo Moro, 11, Piazza Giulio Cesare Bari, Italy; 12 Surgical Research Lab, DRAA-Larkin Health System, Miami, FL USA; 13 Unidad de Cirugía, Universidad de San Martín de Porres, Lima, Peru; 14 Peruvian Society of Robotic Surgery, Lima, Peru; 15 Department of Metabolic and Bariatric Surgery, Metropolitan Hospital, Athens, Greece; 16 Division of Surgery, School of Medicine, European University Cyprus, Nicosia, Cyprus; 17 Department of Minimally Invasive Digestive Surgery, Antoine-Beclere Hospital, Paris-Saclay University, Clamart, Paris, France; 18 Bariatric, Metabolic & Endocrine Unit, General & Digestive Surgery Department, Fuenlabrada University Hospital, Rey Juan Carlos University, Madrid, Spain; 19 Servicio de Ginecología Oncológica, Hospital Pacífica Salud, Pacific Boulevard, Blvd, Pacífica, Panamá; 20 Department of Surgical Oncology, HCG Manavata Cancer Centre, Nashik, 422001 India; 21 General Minimally Invasive (Laparoscopic and Robotic) Surgery Department, Centre Hospitalier de Luxembourg, Luxembourg, Luxembourg; 22 Robotic Surgery Program, Qatar University, Doha, Qatar; 23 Virginia CommonWealth University (VCU), Richmond, VA USA; 24 The View Hospital-Affiliated with Cedars Sinai, Doha, Qatar; 25 Department of Advanced Laparoscopic Surgery, Gloria Patricia Pinzón Clinic, Florencia, Caquetá Colombia; 26 TROGSS - The Robotic Global Surgical Society, Saint Jean de La Ruelle, Orléans, 45140 France; 27 Bariatric Surgery, Specialty Hospital, Western Medical Center, Mexican Social Security Institute (IMSS), Guadalajara, Mexico; 28 The Operating Room Global (TORG), Limerick, Ireland; 29 University of Limerick (UL) Ireland, UL Hospitals, HSE Mid-West, Limerick, Ireland; 30 Bakhutashvili Institute of Medical Biotechnology, Tbilisi State Medical University, Tbilisi, Georgia; 31 Department of Surgery, University Medical Center OWL-Campus Hospital Lippe, 32756 Detmold, Germany; 32 Department of Surgery, CHRISTUS Trinity Clinic, San Marcos, TX USA; 33 Digestive Health Institute, AdventHealth Tampa, Tampa, Florida USA; 34 Minimally Invasive Surgery/General Surgery, New York City (NYC) Health + Hospitals/Harlem, New York, NY USA; 35 Department of General, Visceral and Thoracic Surgery, Sana Clinic Hof, Hof, Germany; 36 Department of Surgery, Centre Hospitalier Universitaire d’Orléans, Orléans, France; 37 Division of General, Mininvasive and Bariatric Surgery, University of Campania “Luigi Vanvitelli”, Naples, Italy; 38 Crouse Health, Syracuse, NY 13210 USA; 39 Department of Surgery, Nacogdoches Medical Center, Nacogdoches, TX USA; 40 University of Houston Tilman J. Fertitta Family College of Medicine, Houston, TX USA; 41 Sam Houston State University College of Osteopathic Medicine, Conroe, TX USA

**Keywords:** Robotic surgery, Human factors, Surgical education, Surgical assessment, Teamwork, Teleoperation, artificial intelligence

## Abstract

Robotic surgery can concentrate control of several technical functions at the console, but such concentration does not itself confer clinical independence, reduce dependence on the perioperative team, or improve patient outcomes. We define surgeon-controlled operative polyphony as the phase-bounded coordination of multiple partially independent technical control streams by one console operator within a distributed perioperative system. This critical narrative review and conceptual synthesis asks which technical functions can be concentrated coherently, where human-factors and team dependencies set limits, and how evaluation, training, and governance should respond. The piano is used as a question-generating model, not as evidence of clinical benefit. PubMed/MEDLINE was searched from inception through 18 August 2026; targeted engineering, educational, neuroscientific, human-factors, and musicological searches and backward citation tracking supplemented retrieval. To analyze this control configuration, we propose the PIANO framework: Phase-specific control, Interface fidelity, Agency allocation, Neuromotor integration, and Orchestrated teamwork. Its scientific novelty lies in linking this control architecture to the PIANO framework: Phase-specific control, Interface fidelity, Agency allocation, Neuromotor integration, and Orchestrated teamwork. PIANO is an analytic and hypothesis-generating framework, not a score or credentialing standard. Concentrated control may shorten selected action loops, but any benefit depends on feedback, workload, shared situation awareness, explicit authority, and rescue capacity. The Competency-Based Assessment of Robotic Surgery Skills (CARS) curriculum provides initial validation evidence for domain-specific assessment of performer competence; repeated cases and use-specific evidence remain necessary for high-stakes decisions. Artificial intelligence (AI) may support phase recognition, feedback, assessment, and personalised training within a human-in-the-loop system. One surgeon may coordinate many operative voices; safe surgery remains a collective, patient-centered performance.

**Supplementary Information:**

The online version contains supplementary material available at https://doi.org/10.1007/s11701-026-04035-8.

## Introduction

This critical narrative review and conceptual synthesis examines a defining organizational feature of robotic surgery: one console surgeon may coordinate visualization, exposure, traction and countertraction, dissection, energy, and suturing through a common interface. It asks three questions: which technical functions can coherently be concentrated under console-surgeon control; where human-factors and team dependencies limit that concentration; and how evaluation, training, and governance should respond [1, 2].

The scientific novelty is not the piano analogy alone. It is the concept of phase-bounded coordination of multiple technical control streams and the PIANO framework as a multilevel human-factors model for deciding when those streams can be concentrated safely and coherently. The pianoforte supplies a bounded vocabulary for independent voices coordinated under one intention, while the framework translates that vocabulary into observable questions about task, interface, authority, operator integration, and teamwork [3, 4].

The boundary must be explicit. A robot may be governed from one console; an operation is not performed by one person. Anesthesiologists, nurses, bedside assistants, technicians, and the wider perioperative system remain indispensable. The patient is neither a score nor an instrument, but the moral center of the operation. The piano is therefore a question-generating model, not evidence that robotic surgery is safer, more efficient, or clinically superior.

We define surgeon-controlled operative polyphony as phase-bounded coordination of multiple partially independent technical control streams by one console operator within a distributed perioperative system. A stream has a distinguishable goal, state, and input, such as visualization, exposure, tissue manipulation, or energy delivery. Polyphony is not inferred from arm count or simultaneity; the streams must be coordinated purposefully during a defined operative phase.

The proposed difference from open or laparoscopic surgery is one of control architecture and degree, not categorical superiority. PIANO - Phase-specific control, Interface fidelity, Agency allocation, Neuromotor integration, and Orchestrated teamwork - tests when consolidation may shorten a control loop and when it may instead increase workload, obscure authority, or weaken collective rescue.

## Methods

### Review design and questions

This is a critical narrative review and conceptual synthesis. The design was chosen to integrate historical, engineering, neuroscientific, educational, human-factors, and clinical literature whose methods and units of analysis are too heterogeneous for pooled estimation. It is not presented as a systematic review and does not claim exhaustive coverage. Reporting and self-appraisal were informed by the six domains of the Scale for the Assessment of Narrative Review Articles (SANRA), which is a critical-appraisal scale rather than a reporting guideline [5]. The synthesis addressed three review questions: which technical functions can coherently be concentrated under console-surgeon control; where human-factors and team dependencies limit that concentration; and how those limits should shape evaluation, training, and governance.

### Information sources and search strategy

PubMed/MEDLINE was the principal biomedical database and was searched from inception through 19 July 2026 without a lower date limit. The five prespecified concept blocks were rerun as a date-limited update from 20 July through 18 August 2026. Searches combined terms for robotic or robot-assisted surgery and teleoperation with: (1) control architecture and human factors; (2) concentrated control, bedside work, and supervision; (3) interface, haptics, and neuromotor integration; (4) training, assessment, and credentialing; and (5) automation, autonomy, artificial intelligence, and governance. Backward citation tracking was performed from key reviews, consensus reports, and foundational studies. Musicological searches covered Cristofori, the pianoforte action, escapement, pedalling, the player piano, and piano pedagogy; neuroscience searches combined piano or musician terms with motor control, finger individuation, and neuroplasticity. Searches were iterative because terminology differs across disciplines. Two authors (LM and FPP) performed source selection and evidence charting. The information-source map and complete query blocks are provided in Online Resource 1, Tables S1 and S2. Record counts are not presented because purposive, iterative sampling was used rather than an exhaustive screening workflow.

### Eligibility, appraisal, and evidence handling

English-language empirical studies, evidence syntheses, consensus or curriculum reports, foundational human-factors and engineering papers, and authoritative historical sources were eligible when they informed at least one review question. Promotional device descriptions, unsupported opinion, and reports without a transferable implication for control, feedback, training, teamwork, assessment, or autonomy were excluded. Public program material was used only for horizon-scanning and never as evidence of validity or effectiveness. Sources were classified as empirical research, evidence synthesis, consensus or curriculum work, foundational theory, historical scholarship, or public programme material. The eligibility criteria, design-specific appraisal questions, and limits placed on inference for each source class are detailed in Online Resource 1, Table S3.

The extended evidence chart, including the source design, principal observation, supported inference, and explicit inferential boundary, is provided in Online Resource 1, Table S4. For evidence supporting a central claim, the study design, unit of observation, unit of inference, principal finding, supported interpretation, and inference boundary were charted. A single cross-study risk-of-bias score would have been misleading because a historical monograph, a human-factors theory, a small feasibility study, and a validation study do not share the same epistemic purpose. Appraisal was therefore design-specific: feasibility studies were not allowed to support effectiveness; known-groups differences were not treated as predictive validity; reliability was separated from validity; edited-video studies were not extrapolated to whole-case or patient outcomes; and public programme descriptions were not treated as research findings.

### Framework derivation and units of analysis

Framework development used an explicit five-stage audit trail. First, the candidate phenomenon was defined and separated from neighbouring ideas such as simultaneity, staffing reduction, clinical independence, automation, and autonomy. Second, recurring control problems were extracted from the literature: phase dependence, fidelity of the perception–action loop, allocation and transfer of authority, operator integration and interference, and distributed team cognition. Third, each problem was mapped to a distinct analytical level. Fourth, candidate domains were retained only when they answered a review question, could be linked to observable data, and generated a proposition that could fail. Fifth, negative cases were used to test boundaries, for example, a configuration in which adding direct console control shortens a local action loop but worsens team awareness or rescue. The complete framework-development audit trail, including conceptual anchors, rejected alternatives, negative cases, and candidate observable data, is provided in Online Resource 1, Table S5.

The conceptual groups were established before the labels Phase-specific control, Interface fidelity, Agency allocation, Neuromotor integration, and Orchestrated teamwork were assigned. The acronym is therefore a mnemonic, not a domain-selection rule. Domains were revised to reduce overlap and to specify a control stream within a defined operative phase as the primary unit of observation. A phase observation is nested within a case, performer, team, platform, and institution. Inferences about competence, safety, or patient benefit consequently require repeated and appropriately sampled observations at the level claimed; a phase-level signal cannot be promoted automatically to a surgeon-level or patient-level conclusion.

PIANO has not undergone multistakeholder content appraisal, consensus, coding-reliability testing, construct testing, or prospective evaluation. It is a sensitizing and hypothesis-generating framework, not an exhaustive taxonomy, causal model, numerical score, credentialing standard, or claim that concentrated control is superior.

### Quantitative data and statistical analysis

No participant-level or study-level quantitative dataset was assembled, and no new meta-analysis, effect-size pooling, hypothesis testing, imputation, missing-data analysis, or sensitivity analysis was performed. The review therefore has no study population, intervention comparator, prespecified clinical endpoint, denominator, or confidence interval of its own. Sample sizes, estimates, confidence intervals, and p values are reported only when they delimit a cited source-level claim; they were checked against the corresponding primary report.

### From clinical observation to a testable construct

The question arose from moving between a piano bench and a robotic console. Both settings require an intention to pass through a complex mechanism and become a precise action in time, but the useful resemblance is functional independence within coordinated intention. At the console, camera, exposure, traction, dissection, energy, and pedals can be coordinated by one operator while remaining nested in a team. This observation generates testable questions about control concentration, workload, feedback, agency transfer, and rescue; it does not establish equivalence or benefit [2, 6].

## From metaphor to model: human-factors foundations

PIANO is grounded in established human-factors constructs. Multiple-resource theory predicts interference when concurrent tasks compete for common perceptual, cognitive, or response resources [7]. Direct camera or retractor control may remove verbal translation and delay, yet an additional visual mode or pedal mapping may create a new source of interference. More control is therefore a hypothesis, not an assumed benefit.

Situation awareness requires perception of relevant cues, comprehension of their meaning, and projection of what may happen next [8]. Console immersion can enrich focal visual information while attenuating cues from the room. The relevant outcome is not command speed alone, but whether the operator and team continue to understand the state of the whole system. Distributed cognition provides the system-level correction: information and action are carried across people, artefacts, representations, and procedures [9]. Moving a function to the console changes what others monitor, anticipate, communicate, and rescue. Automation similarly reallocates work across information acquisition, analysis, decision selection, and action implementation rather than simply subtracting a human task [10].

These theories give PIANO its multilevel architecture. Phase-specific control concerns the task; Interface fidelity the perception-action loop; Agency allocation authority and handover; Neuromotor integration the operator; and Orchestrated teamwork the distributed system. The domains are coupled but not a causal sequence, and benefit must be tested with technical, workload, team, and patient-relevant outcomes.

## The PIANO framework for surgeon-controlled operative polyphony

PIANO translates surgeon-controlled operative polyphony into a provisional multilevel framework for technology assessment, education, and research. Its principal contribution is to analyze the coherence of a control configuration during a defined operative phase rather than to celebrate a device feature or count the number of arms. Control may be coherent during elective dissection and brittle during stapling, hemorrhage, or recovery from a fault. The domains are prompts for observation and hypothesis formation, not items to be summed, and their presence does not imply benefit. Table 1 summarizes the derivation and boundaries of the five domains.

**Table 1:** Derivation matrix for the PIANO domains

Recurring problem and conceptual anchor	Retained domain and analytical level	Reason for retention	Boundary or negative case
Control configuration changes across tasks and phases [2, 6, 11–14]	P — Phase-specific control (task/phase)	Makes the control stream in a defined phase observable and prevents whole-procedure generalization	A function that helps during stable dissection may hinder stapling, bleeding control, or fault recovery
Intention is transformed by an incomplete perception–action loop [7, 8, 15–17]	I — Interface fidelity (human–machine loop)	Separates meaningful feedback and transparent mode state from arm count or degrees of freedom	More available commands can worsen performance when force, state, uncertainty, or failure is obscured
Authority can move among people and automated functions [10, 18–25]	A — Agency allocation (authority/governance)	Requires explicit permission, initiation, veto, handover, rescue, and accountability	A technically capable function remains unsafe when override or recovery rights are ambiguous
Hands, feet, gaze, prediction, and attention compete and integrate [7, 26–28]	N — Neuromotor integration (operator)	Links coordinated motor control to workload, mode awareness, and error recovery	Smooth or fast movement may coexist with tunnelling, mode error, excessive force, or fragile performance
Cognition and rescue are distributed across the perioperative system [2, 9, 29–34]	O — Orchestrated teamwork (team/system)	Prevents concentrated console control from being mistaken for clinical independence	A local console gain is not a system benefit if it increases hidden workload or delays rescue elsewhere

The five conceptual groups preceded the PIANO labels. The acronym is a mnemonic and was not used to select or force domains

**Fig. 1:**
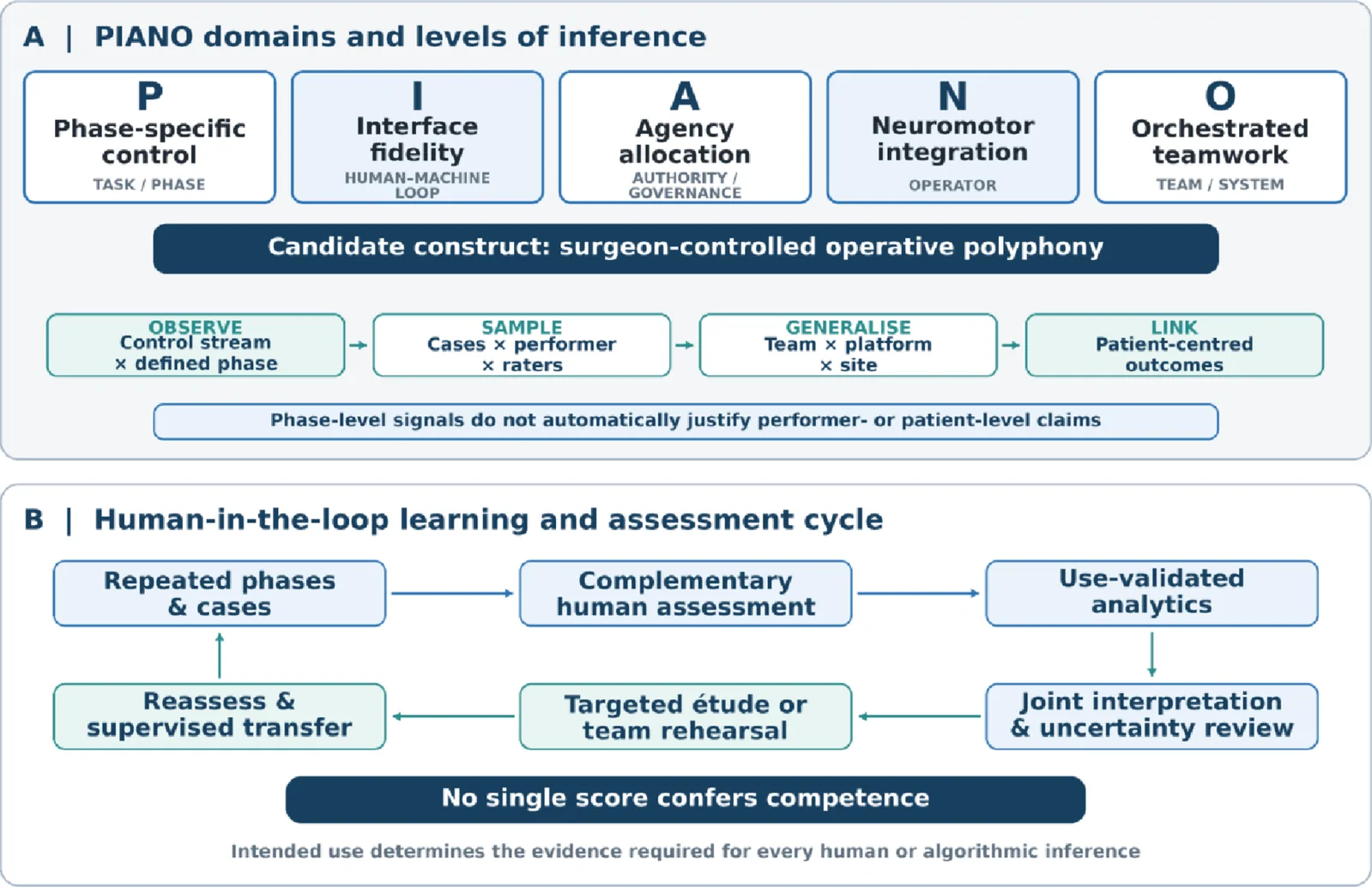
The PIANO domains, levels of inference, and human-in-the-loop learning cycle. Panel A maps the five domains to distinct but coupled analytical levels: a control stream is observed within a defined phase and nested within a case, performer, team, platform, institution, and patient-centered system. Panel B links repeated performance sampling to complementary human assessment, analytics validated for the intended use, joint interpretation, targeted practice or team rehearsal, reassessment, and supervised clinical transfer. No single score confers complete clinical competence, and every inference must be supported at the level and for the use claimed

Table 2 presents operational definitions, candidate observable indicators, and a discriminating question for each domain. The domains occupy different levels but remain coupled: phase-specific control may fail when interface state is opaque; a fluent interface may still obscure responsibility; and smooth neuromotor performance may coexist with attentional tunnelling. PIANO should therefore be applied across phases and contingencies, not completed once as a device checklist. Figure 1 links the domains to the hierarchy of inference and a human-in-the-loop learning cycle.

**Table 2:** The multilevel PIANO framework for surgeon-controlled operative polyphony

Domain and analytical level	Operational definition	Candidate observable indicators	Discriminating question
P — Phase-specific control (task/phase)	Direct coordination by one console operator of two or more distinguishable technical streams required for a defined operative phase	Control-state logs; request-to-action latency; camera and exposure stability; instrument conflict; idle and recovery time	Which streams should be consolidated, shared, automated, or left at the bedside in this phase and condition?
I — Interface fidelity (human–machine loop)	Fidelity and transparency with which intention becomes action and consequence returns to the operator	Input–output mapping; visual, force, or multimodal feedback; mode errors; clutching burden; detection and recovery latency	Does the interface shorten the loop without hiding state, force, uncertainty, or failure?
A — Agency allocation (authority/governance)	Explicit distribution of permission, initiation, veto, handover, supervision, rescue, and accountability among humans and automated functions	Control transfers; override rights; handover time; automation state; escalation and rescue pathways	Who or what may act, under which conditions, and who can recognise, stop, or recover a failure?
N — Neuromotor integration (operator)	Coordinated use of hands, feet, vision, attention, and predictive control across concurrent streams	Bimanual and pedal coordination; gaze allocation; workload; mode awareness; error detection and recovery	When does fluent integration become shared-resource interference, tunnelling, or fragile speed?
O — Orchestrated teamwork (team/system)	Alignment of distributed expertise and situation awareness around a shared patient-centered plan	Closed-loop communication; shared plan; bedside, nursing, and anaesthesia workload; non-technical skills; flow disruptions	Does consolidated control improve the whole work system or merely relocate delay, workload, or risk?

### Hierarchy of observation and inference

The smallest PIANO observation is a control stream during a defined phase. Several phase observations form a case; cases are sampled within performers and teams; teams work with particular platforms and institutions; and patient outcomes arise at the case and patient level. This hierarchy matters. Request-to-action latency may be measured at phase level, workload at operator and team level, flow disruption at team and case level, and complications at patient level. A study that ignores clustering can overstate precision. An assessment that samples one video cannot establish cross-case performer competence. A technical improvement cannot be called a patient benefit without an outcome and design at that level.

For empirical work, repeated phases and cases should be crossed where feasible with raters, performers, procedures, platforms, and sites. Variance attributable to each facet should be estimated before a score is used for comparison or decision-making. Patient-level associations then require prospective case-mix adjustment and sufficient outcome sampling. These are not statistical decorations; they protect against promoting a local signal to a broader claim.

Table 3 applies the five domains to a stable intracorporeal suturing phase and contrasts that configuration with brisk bleeding or platform failure, in which control coherence may be lost.

**Table 3:** Worked application and negative case: stable suturing versus loss of control coherence

PIANO domain	Stable intracorporeal suturing phase	Negative case: brisk bleeding or platform fault
P — Phase-specific control	The console surgeon coordinates the camera, two needle drivers, and stable third-arm exposure around one defined suturing objective	The field is obscured or a device becomes unavailable; maintaining the same number of console-controlled streams no longer serves the immediate rescue objective
I — Interface fidelity	Three-dimensional vision, visible mode state, predictable clutch and pedal mapping, and tissue-deformation cues support graded action	Blood, smoke, occlusion, alarm ambiguity, or degraded control hides consequence or system state and weakens the perception–action loop
A — Agency allocation	The console surgeon controls needle placement; the bedside assistant retains agreed functions for suture passage, suction, exchange, stop, and emergency access	Rescue rights are unclear, an override is delayed, or the console surgeon attempts to retain functions that should transfer immediately to the bedside or another modality
N — Neuromotor integration	Bimanual and pedal actions remain fluent, pauses are deliberate, workload is tolerable, and error recovery is preserved	Attentional tunnelling, mode confusion, excessive force, or inability to disengage indicates that control density has exceeded a safe margin
O — Orchestrated teamwork	The team shares the next steps and contingency plan; changes are announced and closed-loop responses remain intact	Bedside, nursing, or anaesthesia teams cannot anticipate the console surgeon’s state, workload, or rescue need; local control delays collective response

In the negative case, safe performance may require less consolidated control, immediate agency transfer, conversion, or abandonment of the maneuver. This is a boundary of the configuration, not a moral failure of the operator

The example shows why operative polyphony is phase-bounded. During stable suturing, direct coordination may reduce conversational latency and preserve exposure. During hemorrhage or a fault, the same configuration may become brittle. The appropriate response is not to defend the soloist’s control, but to reorchestrate agency around rescue. In technology studies, PIANO directs attention to who controls visualization, exposure, suction, energy, stapling, and emergency actions; how control transfers; what feedback returns; and where workload moves. In education, it separates component technique, integrated phase performance, team participation, and entrustment. In procurement, it tests coherence and recoverability of the human–machine loop. In autonomy research, it requires every gain in machine capability to be paired with an operating domain, uncertainty representation, supervision plan, and route to safe handover. For training, PIANO adds an integration layer to domain-specific technical assessment. A rubric may describe individual control streams; PIANO asks whether they remain coordinated with feedback, workload, distributed agency, and the team. Assessment should inform focused practice and supervised transfer. Contributions to entrustment or credentialing require repeated sampling across relevant variation, calibrated review, and evidence validated for the intended decision.

### Interface fidelity: from intention to action

Cristofori’s early eighteenth-century hammer action allowed key velocity to produce a graduated acoustic consequence; escapement enabled the hammer to strike and fall away [3, 35]. The historical point is limited but useful: expression depended not simply on more keys, but on a more responsive relation between intention, mechanism, and feedback.

Robotic systems transform the surgeon’s input through motion scaling, tremor filtration, articulation, visual alignment, energy integration, and platform-specific control mappings [1, 36–39]. Interface quality therefore depends on how transparently intention becomes action and how clearly system state, consequence, uncertainty, and failure return to the operator.

Many platforms provide rich vision but limited direct force feedback. Tissue tension is inferred from deformation, instrument behaviour, and experience. Reviews suggest that force or haptic feedback may help selected tasks, but effects depend on task, modality, fidelity, and operator experience [15–17]. An updated cross-domain systematic review likewise found task-dependent benefits and persistent barriers to clinical translation [40]. More commands or degrees of freedom do not necessarily produce better control; the complete perception-action loop is the relevant unit.

### Phase-specific control: coordinated technical streams

A pianist can coordinate distinct voices with different rhythms and priorities under one musical intention [41]. At a robotic console, a bounded parallel exists when vision, exposure, traction, dissection, energy, and suturing retain distinguishable goals and states but are coordinated by one operator during a defined phase. This is the core of surgeon-controlled operative polyphony.

The potential advantage is not a larger number of simultaneous actions or a smaller team. It is a shorter and more faithful control loop for selected functions. Direct camera or third-arm control may reduce repeated verbal translation and maintain stable exposure, but only when usability, feedback, workload, and recovery remain coherent [11].

Selected feasibility studies show that camera control and several operative arms can be brought under one console surgeon in bounded workflows [12, 13]. These findings support technical feasibility, not clinical independence, comparative superiority, or reduced rescue requirements. We retain the term ‘solo surgery’ only when reporting the original authors’ terminology.

As control streams accumulate, selection, monitoring, switching, and error recovery also converge. Robotic surgery may reduce some physical demands while introducing control complexity, separation from the room, interruptions, and redistributed workload [2, 6]. In a 2026 single-centre study of 72 supervised colorectal cases involving 10 surgeons, junior surgeons reported higher mental, temporal, task-complexity, and situation-awareness workload than certified surgeons despite comparable short-term clinical outcomes under direct supervision [42]. Work-system interventions such as team training, checklists, and workspace redesign remain less frequently evaluated than the challenges they address [14].

**Fig. 2:**
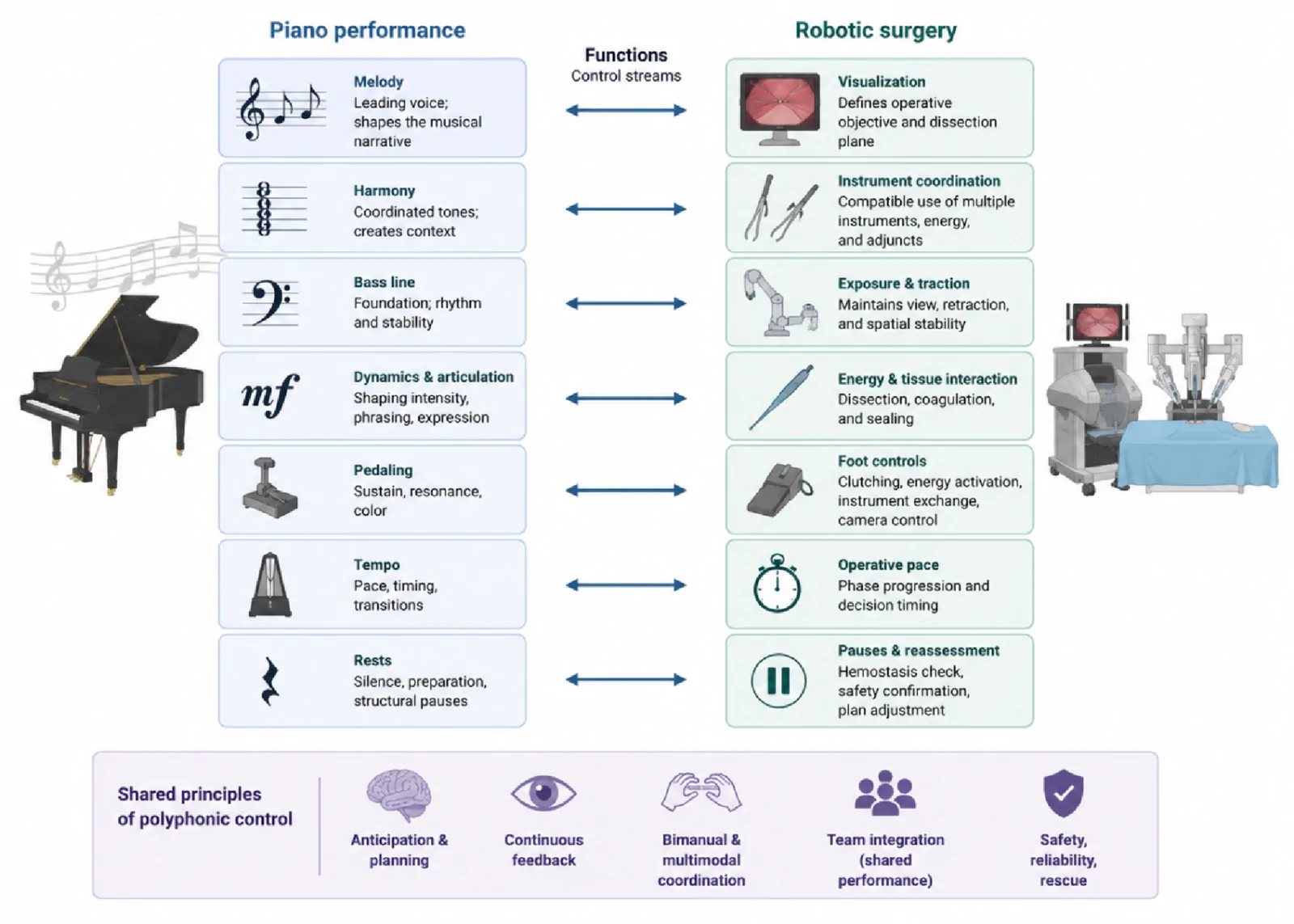
Heuristic mapping between piano performance and robotic surgery. Seven paired control functions and five shared principles illustrate the analogy. It does not imply equivalence, clinical independence, reduced staffing, or clinical benefit

Table 4 maps musical functions to robotic analogues as a heuristic, and Fig. 2 visualises seven paired functions and five shared principles. Neither asserts equivalence or benefit. The empirical question is which streams should be consolidated, shared, automated, or returned to the team in each phase and condition.

**Table 4:** Musical functions and robotic surgical analogues

Piano domain	Robotic surgical analogue	Human-factors implication
Melody	Primary operative objective and active dissection plane	Must remain perceptually dominant despite competing tasks
Harmony	Coordinated use of instruments, imaging, energy, and adjuncts	Components must be mutually compatible and temporally aligned
Bass line	Exposure, retraction, and spatial stability	A stable foundation frees attention for fine action
Counterpoint	Traction, countertraction, and simultaneous tissue handling	Independent movements require integration rather than mere simultaneity
Dynamics and articulation	Motion scaling, pressure, energy modulation, and tissue-specific handling	Control quality depends on graded input and informative feedback
Pedaling	Camera clutching, energy activation, instrument switching, and auxiliary controls	Feet extend control but increase mapping and mode-awareness demands
Tempo	Operative pace and phase transitions	Speed should serve clarity, safety, and anticipation
Rests	Deliberate pause, reassessment, hemostatic check, and team confirmation	Silence and stopping are active safety behaviors

The table is a heuristic map, not a claim of equivalence between artistic performance and patient care

### Neuromotor integration, workload, and fluency

Advanced piano performance depends on finger individuation, bimanual coordination, anticipatory timing, and sensorimotor prediction; trained musicians show experience-related motor and auditory adaptations [26, 27]. This does not imply direct transfer to surgery. It supports the narrower educational proposition that component actions can become integrated through structured practice.

Robotic surgery requires coordinated use of two master controllers, pedals, clutching, instrument exchange, vision, and attention. Fluency may reduce basic control demands, but speed and smoothness can coexist with mode error, attentional tunnelling, excessive force, or fragile recovery. Evaluation should therefore include exposure, instrument conflict, indirect and non-calibrated proxies for tissue–instrument loading, such as instrument kinematics and visually observed tissue deformation, mode awareness, workload, and error recovery rather than completion time alone. Training should make console mechanics reliable enough to free attention for anatomy, strategy, oncological judgment, and early recognition of deviation. This is trained human automaticity, not machine autonomy. Workload varies with approach, expertise, phase, and team configuration [6, 28], so proficiency must be sampled under relevant variation and not inferred from one fluent performance.

### Training, assessment, and competency development

The training analogy is strongest when it remains educational: component techniques are isolated for deliberate practice and then reintegrated into complete performance. Accurate execution is necessary but not sufficient; interpretation, adaptation, and recovery must also be demonstrated.

Robotic curricula similarly progress from camera navigation, clutching, bimanual transfer, energy use, and suturing to compound tasks, procedural phases, complete operations, and supervised entrustment. Reviews and consensus work support structured curricula, proficiency-based progression, and graduated responsibility beyond case counts [43–45]. A 2026 systematic review of technology-enhanced basic robotic training included 17 studies and 484 participants but found evidence concentrated at learning outcomes, with no workplace-behavior or patient-level outcomes [46]. A separate meta-analysis of seven trials found imprecise, non-significant pooled effects for global technical skill (Hedges’ g 0.77, 95% CI −0.64 to 2.18) and task completion time (g 0.47, 95% CI −0.35 to 1.29) [47]. Simulation remains a practice environment rather than proof of independent clinical performance.

Assessment tools address complementary constructs. GEARS describes global technical performance [48]; R-OSATS evaluates standardized dry-laboratory tasks [49]; ARCS separates console-manipulation domains [50]; RO-SCORE addresses supervised operative independence [51]; a platform-specific checklist provides preliminary validity evidence for setup and docking [52]; and RAS-NOTECHS examines multidisciplinary non-technical performance [29]. Automated metrics and artificial intelligence (AI) observe the signals available to them. Table 5 compares the intended constructs, evidence, and defensible uses of these approaches [53–56].

**Table 5:** Complementary instruments and data sources for evaluating robotic performance

Instrument or data source	Intended construct and level	Published evidence illustrated here	Defensible use and principal boundary
GEARS [48]	Global technical performance of a console operator	Initial clinical known-groups, internal-consistency, and inter-rater evidence; broad subsequent use summarized in reviews [53, 54]	Useful for global formative ratings across repeated samples; limited procedure detail and no direct measure of teamwork, entrustment, or patient outcome
R-OSATS [49]	Technical execution of five standardized robotic dry-laboratory drills	Multi-institutional study of 105 participants with known-groups and inter- and intra-rater evidence	Useful for standardized task training and benchmarking; does not establish whole-procedure or workplace competence
ARCS [50]	Six distinct console-manipulation skills	Three blinded raters scored three standardized tasks performed by 15 participants across experience groups	Useful for diagnostic feedback on console mechanics; initial evidence is based on a small, model-task sample rather than varied clinical cases
RO-SCORE [51]	Supervised independence and intraoperative entrustment	Pre/post cohort of 31 robotic-naïve residents after simulator training, with no concurrent control group	Useful for workplace feedback on required supervision; ratings are case-, supervisor-, and context-dependent and are not a pure technical score
CARS/CARS 2.0 [57, 58]	Ten domain-specific robotic activities, including seven assessable from operative video	Initial CARS differentiated resident training levels in person and by blinded video and showed moderate inter-rater reliability in analyzed general, abdominal wall reconstruction, colorectal, and metabolic and bariatric surgery videos; CARS 2.0 extended competency-oriented scoring to 320 international raters across seven video-visible domains	Supports formative and competency-oriented assessment of domain-specific performer competence; repeated representative cases and use-specific validation remain necessary for high-stakes credentialing
RAS-NOTECHS [29]	Multidisciplinary non-technical performance at team level	Preliminary validity and reliability evidence in robotic operating-room settings	Complements technical assessment; cannot substitute for direct evaluation of console or procedure-specific skill
Automated performance metrics and AI [53–55]	Observable kinematic, event-log, video, or multimodal features	Heterogeneous retrospective and prospective studies, often with restricted tasks and samples	Useful as an adjunct when validated for the intended task, population, and decision; an algorithmic output is not itself a competence or credentialing decision
Setup and docking checklist [52]	Platform-specific setup and docking performance	Pre/post study of 51 novices; total-score inter-rater ICC 0.91 (95% CI 0.75–0.97) across 12 videos scored by three blinded raters	Supports structured formative assessment on the da Vinci Xi platform; preliminary, single-course evidence is insufficient for accreditation or cross-platform inference

Validity concerns the interpretation and use of scores in a specified context. Evidence for one purpose, task, population, or sampling design does not transfer automatically to another [56]

#### CARS as a competency-oriented assessment framework

The Competency-Based Assessment of Robotic Surgery Skills (CARS) curriculum is especially relevant because its ten domains represent discrete components of robotic technical performance. The initial single-center study evaluated 29 residents in person and included blinded scoring of 20 de-identified operative videos with CARS and GEARS. CARS differentiated beginner, intermediate, advanced resident, and attending (consultant-level) performance in the sampled settings; inter-rater reliability was moderate in the analyzed inguinal-hernia videos [57]. These results provide initial validation evidence for competency-based assessment of domain-specific performer competence.

CARS 2.0 expanded the curriculum through a global, blinded video-assessment study. Three hundred and twenty respondents across training stages and specialties rated a common edited robotic case using seven video-visible CARS domains: dissection, tissue handling and retraction, stapler use, arm exchange, endoscope use, suturing and knot tying, and wristed articulation [58]. Port placement, docking, and ergonomics were appropriately designated not assessable from the video.

Scores differed across all seven visible domains according to evaluator experience, demonstrating global feasibility and sensitivity of competency-oriented scoring to rater background. Together with the initial CARS study, these findings support CARS as a structured framework for evaluating domain-specific robotic performer competence and for identifying targets for training. They also emphasize the need for rater calibration when scores inform comparison or progression.

The positive appraisal remains use-specific. Initial CARS validation supports formative and competency-oriented interpretation within sampled tasks and settings; CARS 2.0 adds response-process, deployment, and generalizability evidence. Repeated representative cases, calibrated raters, cross-platform testing, longitudinal progression, and consequences analysis are still required before a CARS score is used alone for high-stakes credentialing or prediction of patient outcomes [56]. This boundary strengthens rather than negates the validation argument.

Within PIANO, CARS supplies domain-specific evidence about several technical control streams, while the wider framework asks whether those streams remain integrated with feedback, workload, authority, teamwork, and rescue. CARS can therefore contribute directly to assessment of performer competence without being asked to represent every dimension of clinical independence.

CARS 3.0 is an emerging research-and-development curriculum involving simulation, video-data platforms, AI, and human judgment [59, 60]. No peer-reviewed validation report was identified by the review cut-off. Future evaluation should predefine intended uses, document data provenance and case mix, quantify reliability and uncertainty, test external performance and subgroup fairness, and examine educational and clinical consequences.

### Artificial intelligence and personalized training across PIANO

AI can contribute across all five PIANO domains: workflow and phase recognition for Phase-specific control; context-aware feedback and interface monitoring for Interface fidelity; shared-autonomy and handover analysis for Agency allocation; kinematic, gaze, indirect tissue-loading-proxy, and workload analysis for Neuromotor integration; and communication, workflow, and team-pattern analysis for Orchestrated teamwork.

These are candidate functions whose value must be validated for a declared task and decision, not assumed from model accuracy alone [53–55, 61]. For example, one kinematics benchmark reported 97.1% pooled leave-one-subject-out accuracy, while off-task transfer fell to 61–95%, illustrating why internal benchmark performance cannot establish clinical generalization [62].

Multimodal assessment also creates a route to personalized training. Repeated cases can reveal whether an individual struggles with camera control, tissue handling, instrument coordination, anticipation, workload, or error recovery. Validated analytics may identify patterns, while faculty interpret them, prescribe targeted practice or team rehearsal, and decide when supervised transfer is appropriate. Dual-console teaching can support direct observation and control transfer, but its value still depends on communication and graduated entrustment [63, 64].

Technical competence is one component of complete surgical competence. Judgment, adaptation, complication management, communication, leadership, and teamwork must also be demonstrated across procedures, phases, complexity, unexpected events, and clinical environments. CARS can provide validated domain-specific evidence of performer competence; no single technical score, simulator result, case count, or algorithm can independently confer unsupervised clinical autonomy.

### Orchestrated teamwork: the operator is not solitary

Robotic surgery remains the work of a distributed system. Bedside colleagues dock and exchange instruments, manage suction and stapling, introduce materials, respond to collisions or faults, and retain immediate access to the patient. Anesthesia and nursing teams maintain physiology, equipment, sterility, supplies, and anticipation. Whatever the surgeon controls directly is nested within collective competence.

Robotics changes proximity, communication, shared mental models, and professional roles [2, 30]. A scoping review of 54 empirical studies grouped evidence around communication, non-technical skills, flow disruption, and education while noting inconsistent definitions [31]. RAS-NOTECHS supports observation of team-level non-technical performance [29]. Console skill and collective performance must be evaluated together but remain distinct outcomes.

The bedside assistant is indispensable during bleeding, instrument failure, stapling, specimen handling, emergency access, and tasks the platform cannot perform. Evidence identifies technical, anticipatory, and non-technical competencies specific to the role, and assistant experience may influence performance early in the console surgeon’s learning curve [32–34]. A 2026 systematic review found only three comparative observational studies of physician versus non-physician bedside models; possible efficiency benefits were reported, but safety evidence remained heterogeneous and confounded [65]. Interprofessional emergency-undocking simulation has shown feasibility and improved readiness in a 30-participant, 12-cycle study, without establishing patient benefit [66]. Concentrated console control changes this expertise; it does not eliminate it.

The terminology must reflect these different levels of control:

- A technical control stream is a function with a distinguishable goal, state, and control input, such as visualization, exposure, tissue manipulation, or energy delivery.
- Consolidated console control means that selected technical control streams are directly governed by one console operator during a defined phase within a staffed perioperative system.
- Clinical independence would mean that the surgeon can safely provide the entire perioperative act alone; this is neither the reality nor the goal.
- Machine automation means that a defined function is executed according to encoded rules with limited real-time human input.
- Machine autonomy refers to a system’s capacity to perceive, decide, and act across a specified domain, at a stated level of supervision.
- Distributed operative agency describes how authority and action move among the console surgeon, bedside team, anesthesia team, software, and hardware over time.

These distinctions preserve the useful claim: a robotic surgeon can coordinate a richer technical texture without owning the entire clinical system. Consolidated console control and clinical independence are not synonyms.

Technical performance has value only when it serves patient safety, oncological or procedural quality, recovery, access, and outcomes that matter to the patient. Any assessment or automation strategy must therefore preserve patient primacy, collective rescue, and accountable human judgment.

### Agency allocation, automation, and artificial intelligence

Automation and autonomy must be described by function and level. They may affect information acquisition, analysis, decision selection, or action implementation under different degrees of human supervision [10, 18, 19]. Most surgical robots cleared by the United States Food and Drug Administration (FDA) between 2015 and 2023 remained at low levels of autonomy [20].

Experimental systems have completed supervised autonomous soft-tissue subtasks and intestinal anastomosis under controlled conditions [21, 22], while surgical data science may support phase recognition, decision support, error prediction, and automated subtasks [23, 24]. These achievements do not establish autonomous management of an operation or patient.

Each automated function should declare its operating domain, required conditions, uncertainty, handover plan, failure modes, override rights, and accountable supervisor. The IDEAL framework for surgical robotics similarly emphasizes staged development, comparative evaluation, transparency, and long-term monitoring [25]. PIANO locates these requirements within Agency allocation while retaining their consequences for interface, operator, and team.

### Empirical hypotheses and disconfirming observations

The following propositions make predictions about the world. Each can be contradicted and should be revised if the predicted pattern is not observed.

- Control-loop hypothesis. Consolidating a phase-relevant technical stream will reduce request-to-action latency and translation loss only when interface fidelity and feedback are adequate. Within-phase comparative or high-fidelity crossover studies should measure latency together with error, workload, and recovery. No latency reduction, or an increase in error or recovery time, would disconfirm benefit for that configuration.
- Control-density hypothesis. The relationship between the number and complexity of directly controlled streams and performance will be non-linear. Experimental manipulation of control density should reveal a point beyond which shared-resource interference impairs situation awareness or recovery. Persistent monotonic improvement without a workload or safety cost across the tested range would require revision of the hypothesis.
- Work-redistribution hypothesis. Removing a bedside request will redistribute rather than necessarily eliminate work. Paired console, bedside, nursing, and anaesthesia measures should identify where monitoring, communication, and rescue work move. Demonstration that work genuinely disappears without compensatory demand or loss of awareness would narrow this claim.
- Multilevel-assessment hypothesis. Video, kinematics, and event logs will explain only part of integrated robotic performance. Their incremental value should be tested across repeated cases, performers, raters, teams, and external sites against outcomes appropriate to the intended use. Robust cross-case and cross-site prediction of integrated performance by one modality alone would disconfirm the proposed need for triangulation in that context.
- Automation-work hypothesis. Function-level automation will alter supervisory, coordination, and handover work even when manual demand falls. Studies should measure mode awareness, intervention, handover, and recovery as well as task success. Reliable benefit without new monitoring or coordination cost within a declared operating domain would justify moving agency and narrowing this prediction.

### Normative safety principles

The framework also contains principles that are not empirical hypotheses and should not be disguised as such. They express the conditions under which evidence ought to be interpreted and action governed:

Patient primacy: concentrated control has value only through patient-relevant benefit; virtuosity is not an independent endpoint.

Proportionate agency: control should be consolidated, shared, automated, or transferred according to competence, context, risk, and recoverability—not according to technological novelty or professional ownership.

Transparent authority: mode state, uncertainty, override, handover, rescue, and accountability must be explicit before a function is deployed.

Use-specific validity: evidence must support the exact assessment interpretation and decision proposed; formative feedback, entrustment, credentialing, and patient-safety surveillance require different arguments.

System and equity accounting: a local ergonomic or efficiency gain is not a system benefit if it transfers hidden workload, weakens access, or distributes error unequally.

Separating these principles from the hypotheses prevents a category error. Data can test whether a configuration shortens latency or redistributes workload. Data inform, but does not alone create, the obligation to protect the patient, preserve rescue, or make accountability visible.

### Limits of the analogy and a research agenda

The analogy is useful only while its limits remain visible. The piano belongs to a particular Western technological and musical history and cannot stand as a universal model of dexterity or collaboration. Other traditions may offer better accounts of improvisation, distributed attention, or ensemble intelligence. Musical success is aesthetic and permits plural judgments; surgery is constrained by biology, evidence, ethics, regulation, and the patient’s preferences. The motor worlds also differ. Tissue is deformable, variable, and vulnerable in ways that keys and strings are not, and the robotic surgeon often works through incomplete sensory mediation. Finally, “solo” is an ordinary and honorable word in music. In surgery it can conceal indispensable labor.

The methodological limits are substantive. PubMed/MEDLINE was the principal biomedical database, while other disciplinary searches were targeted and iterative. There was no registered protocol, exhaustive record accounting, dual screening, duplicate extraction, or formal study-level risk-of-bias assessment. The detailed information-source mapping, reproducible search queries, appraisal rules, framework-development audit trail, and extended evidence chart provided in Online Resource 1, Tables S1-S5 improve transparency and reproducibility; they do not convert this critical narrative review into a systematic review. The design can generate and discipline a conceptual explanation, but it cannot establish prevalence, comparative effectiveness, or causal benefit.

PIANO itself has not been validated yet. A credible development sequence should begin with qualitative appraisal by surgeons, anaesthetists, nurses, bedside assistants, engineers, educators, human-factors researchers, patients, and ethicists, deliberately seeking missing constructs and negative cases. Revised definitions could support a phase-based observational manual. Feasibility and inter- and intra-rater reliability should precede construct testing against device logs, interface events, workload, situation awareness, technical performance, teamwork, and flow disruption. Multicenter studies should then test generalization across procedures, platforms, experience levels, institutions, and resource settings. Only after that should associations with learning, entrustment, rescue, and patient-relevant outcomes be examined. If a numerical score is ever proposed, it will require a separate, use-specific validity argument and consequences analysis [56]. Until then, PIANO remains a language for inquiry rather than a score for judgment.

Six linked research programs follow:

- Establish content and response-process evidence. Multistakeholder concept elicitation, cognitive interviewing, and Delphi work should test whether the five domains are comprehensible, distinct, sufficient, and applicable across specialties and cultures. Stakeholders should be asked specifically for absent concepts and cases in which the framework fails.
- Build and test phase-level observation. A coding manual should define control streams, phase boundaries, agency transfers, mode errors, and rescue events. Reliability, rater calibration, sampling requirements, and measurement invariance across platforms and specialties should be quantified before aggregation.
- Test control density and interface fidelity experimentally. Time-synchronized video, device logs, gaze, workload, interface state, tissue-effect surrogates, and recovery performance can identify when consolidation shortens a control loop and when resource interference reverses the gain.
- Measure redistribution and resilience across the whole team. Console effects must be paired with bedside, nursing, and anesthesia workload, communication, shared situation awareness, flow disruption, fault recovery, and emergency conversion. Stable elective phases and deteriorating contingencies should both be sampled.
- Validate learning, assessment, AI, and automation by intended use. Comparative longitudinal studies should test whether the PIANO learning cycle improves transfer beyond case accumulation or undifferentiated feedback. Emerging programs, including CARS 3.0, should predefine claims; report provenance, annotation, reference standards, uncertainty, case mix, external performance, subgroup fairness, override, and failure recovery; and evaluate educational or clinical consequences before high-stakes use.
- Link system benefit to patients and equity. Claims about control or automation must ultimately be related to safety, access, recovery, functional outcome, oncological quality where relevant, resource use, distributional equity, and the patient’s experience of care. Patient and public partners should help determine which outcomes justify technological complexity.

This agenda makes the concept corrigible. If the domains cannot be distinguished, they should be merged or discarded. If consolidated control improves a local technical measure while damaging team awareness or rescue, it is not a system benefit. If a shared or automated function performs better within a declared domain and transfers safely, agency should move. PIANO is not an argument for maximizing surgeon control; it is a disciplined way to ask when control should be consolidated, shared, automated, or returned to the team.

## Conclusion

Robotic surgery can concentrate several phase-relevant technical control streams under one console surgeon. The central contribution of this review is surgeon-controlled operative polyphony as a testable construct and PIANO as a multilevel framework for analyzing when that control configuration is coherent.

Concentrated control is not clinical solitude and does not guarantee benefit. A shorter console action loop may increase workload or lengthen communication with the room; dexterity may improve while feedback remains incomplete; and a configuration that works during a stable phase may become unsafe during hemorrhage, failure, or overload. Safe performance requires explicit authority, informative feedback, shared awareness, and rapid redistribution of agency.

CARS provides initial validation evidence for domain-specific assessment of robotic performer competence, while PIANO links technical performance to interface, workload, team, and governance. Human and algorithmic assessments should guide personalized practice, expert interpretation, reassessment, and supervised transfer. Prospective multistakeholder and multicenter work is now needed to validate the PIANO domains and test whether the framework improves learning, resilience, or patient-relevant outcomes.

One surgeon may coordinate many operative voices; safe surgery remains a collective, patient-centered performance.

## Supplementary Information

Below is the link to the electronic supplementary material.

Supplementary Material 1

## Data Availability

No datasets were generated or analysed during the current study.
