# Supplementary material for: The robotic PIANO: a critical narrative review of surgeon-controlled operative polyphony, human factors, and robotic training
Source: J Robot Surg. 2026 Oct 7;20(1):1067. doi: 10.1007/s11701-026-04035-8 (PMC13645995; doi:10.1007/s11701-026-04035-8)
Supplement: Supplementary file 1 [file 11701_2026_4035_MOESM1_ESM.docx]

Online Resource 1
The Robotic PIANO: A Critical Narrative Review of Surgeon-Controlled Operative Polyphony, Human Factors, and Robotic Training
Journal: Journal of Robotic Surgery
Authors: Luigi Marano, Mirko Barone, Mehmet Mahir Özmen, Francesco Paolo Prete, Silvia Malerba, Edmundo Inga-Zapata, Panagiotis Lainas, Elena Ruiz-Úcar, Miguel Angel Cáceres Yap, Vikas Jain, Beniamino Pascotto, Juan Santiago Azagra, Salaam Sadi, Adolfo Perez-Bonet, Luis Osvaldo Suárez Carreón, Archil Aladashvili, Miljana Vladimirov, Bojan Jovanovic, Mahmoud Al-Balas, Mario Testini, Cesar Santiago, Sharona Ross, Sharique Nazir, Yogesh Vashist, Adel Abou-Mrad, Rodolfo J. Oviedo, David Albala.

Corresponding author: Prof Francesco Prete, Academic General Surgery Unit 'F. Paccione', Department of Precision and Regenerative Medicine and Jonian Area (DiMePRe-J), University of Bari School of Medicine, Bari, Italy. Email: Francesco.prete@uniba.it

Table S1. Information sources, coverage, and role in the synthesis

| Source | Coverage and final search date | Role and boundary |
| --- | --- | --- |
| PubMed/MEDLINE | Inception to 18 August 2026; no lower date limit; English-language sources selected | Principal biomedical search. Used for empirical studies, reviews, curricula, assessment, team performance, human factors, haptics, and autonomy. Not treated as exhaustive for engineering or musicology. |
| IEEE Xplore and publisher databases | Targeted searches rerun to 18 August 2026 | Used for automation, human-machine interaction, teleoperation, and foundational engineering papers not consistently indexed in MEDLINE. No bibliometric completeness claim. |
| Backward citation tracking | Key reviews, consensus reports, validation studies, and foundational papers | Used to recover seminal or cross-disciplinary sources and to examine the evidentiary lineage of central claims. |
| Musicological books and museum collections | Targeted searches to 18 August 2026 | Used for Cristofori, pianoforte action, escapement, pedalling, player piano, and social history. Historical sources support the musical history only, not clinical effectiveness. |
| Neuroscience searches | Targeted PubMed and publisher searches to 18 August 2026 | Used for musician neuroplasticity, finger individuation, temporal control, and bimanual coordination. Findings were not used to claim that pianists are predisposed to robotic skill. |
| Official congress and programme material | Publicly accessible 2026 programmes [59, 60] | Used only for horizon-scanning of the label CARS 3.0. Programme descriptions were not treated as an instrument, study, or evidence of validity. |

Table S2. Reproducible PubMed/MEDLINE query blocks and targeted cross-disciplinary search terms.

Query 1 - control architecture and human factors

(("Robotic Surgical Procedures"[Mesh] OR "robotic surgery"[tiab] OR "robot-assisted surgery"[tiab] OR "robotic-assisted surgery"[tiab] OR "surgical robot*"[tiab] OR teleoperat*[tiab]) AND ("human factors"[tiab] OR ergonom*[tiab] OR workload[tiab] OR "task load"[tiab] OR "situation awareness"[tiab] OR communication[tiab] OR teamwork[tiab] OR "distributed cognition"[tiab] OR "multiple resource*"[tiab] OR "flow disruption*"[tiab]))

Query 2 - concentrated control, bedside work, and supervision

(("robotic surgery"[tiab] OR "robot-assisted surgery"[tiab] OR "surgical robot*"[tiab]) AND ("solo surgery"[tiab] OR "surgeon-controlled camera"[tiab] OR "camera holder*"[tiab] OR "laparoscope manipulator"[tiab] OR "third arm"[tiab] OR retraction[tiab] OR "bedside assistant*"[tiab] OR "dual console"[tiab] OR proctor*[tiab] OR supervis*[tiab] OR entrust*[tiab]))

Query 3 - interface, haptics, and neuromotor integration

(("robotic surgery"[tiab] OR "robot-assisted surgery"[tiab] OR teleoperat*[tiab]) AND ("haptic feedback"[tiab] OR "force feedback"[tiab] OR tactile[tiab] OR kinesthetic[tiab] OR "motion scaling"[tiab] OR "human-machine interface"[tiab] OR "mode error*"[tiab] OR clutch*[tiab] OR pedal*[tiab] OR bimanual[tiab] OR neuromotor[tiab] OR gaze[tiab]))

Query 4 - training, assessment, and credentialing

(("robotic surgery"[tiab] OR "robot-assisted surgery"[tiab]) AND (training[tiab] OR education[tiab] OR simulation[tiab] OR curriculum[tiab] OR "proficiency-based"[tiab] OR "competency assessment"[tiab] OR "technical skill*"[tiab] OR "video assessment"[tiab] OR credential*[tiab] OR accreditation[tiab] OR GEARS[tiab] OR "R-OSATS"[tiab] OR ARCS[tiab] OR "RO-SCORE"[tiab] OR CARS[tiab] OR "RAS-NOTECHS"[tiab] OR "automated performance metric*"[tiab]))

Query 5 - automation, autonomy, AI, and governance

(("robotic surgery"[tiab] OR "robot-assisted surgery"[tiab] OR "surgical robot*"[tiab]) AND (automation[tiab] OR autonomous[tiab] OR autonomy[tiab] OR "levels of autonomy"[tiab] OR "artificial intelligence"[tiab] OR "machine learning"[tiab] OR "surgical data science"[tiab] OR handover[tiab] OR override[tiab] OR governance[tiab] OR regulation[tiab] OR accountability[tiab]))

Targeted cross-disciplinary terms

- Human factors: "multiple resources", "situation awareness", "distributed cognition", "types and levels of automation", "control allocation", "mode awareness", and "human-automation interaction".
- Musicology: "Bartolomeo Cristofori", "gravicembalo col piano e forte", "pianoforte action", "escapement", "double escapement", "piano pedals", "player piano", "piano pedagogy", and "piano social history".
- Neuroscience: (piano OR pianist OR musician) AND (motor control OR finger individuation OR bimanual coordination OR neuroplasticity OR temporal control).
- Assessment follow-up: exact names and acronyms of GEARS, R-OSATS, ARCS, RO-SCORE, CARS, RAS-NOTECHS, plus "validity", "reliability", "generalisation", "entrustment", "patient outcome", and "consequences".

Table S3. Source eligibility and inference rules

| Source class | Included when | Appraisal question | Inference not permitted without additional evidence |
| --- | --- | --- | --- |
| Empirical clinical or simulation study | Directly informed control, feedback, workload, training, assessment, teamwork, bedside work, or automation | What were the design, sampling frame, unit of observation, comparator, outcome, and setting? | Feasibility was not interpreted as effectiveness; model-task performance was not promoted to whole-procedure competence. |
| Validation or reliability study | Evaluated an instrument or data-derived metric relevant to robotic performance | What interpretation and use were proposed? Which sources of validity evidence and sampling facets were examined? | Known-groups differences were not treated as predictive validity; reliability was not treated as sufficient validity; one task or video was not generalised across cases. |
| Systematic, scoping, or mixed-studies review | Synthesised a central multidisciplinary evidence domain | Were search scope, heterogeneity, and limitations clear? | Review conclusions were not used beyond the studies and outcomes actually synthesised. |

| Source class | Included when | Appraisal question | Inference not permitted without additional evidence |
| --- | --- | --- | --- |
| Consensus or curriculum report | Described training architecture, roles, or expert agreement | Who participated, how was consensus established, and what remained untested? | Consensus was not treated as evidence of educational or patient benefit. |
| Foundational theory | Supplied a human-factors construct needed to generate testable questions | Is the construct conceptually applicable and is the transfer to robotic surgery stated as a hypothesis? | Theory was not treated as direct evidence that a robotic configuration improves outcomes. |
| Historical scholarship | Established the history or mechanics of the piano | Is the source authoritative for the historical claim? | Historical analogy was not used as evidence of surgical safety, efficiency, or superiority. |
| Public programme material | Established that a named programme was publicly described | Does the source document status without making a research claim? | No reliability, validity, fairness, effectiveness, or outcome inference. |

Table S4. Representative evidence, supported inference, and explicit boundary

| Topic | Source, design, and unit of observation | Principal observation | Supported inference | Explicit boundary |
| --- | --- | --- | --- | --- |
| Human-factors foundations | Wickens [7], Endsley [8], Hutchins [9], Parasuraman et al. [10]; foundational theory | Concurrent work competes for resources; situation awareness is vulnerable; cognition and automation are distributed across functions and agents | Supports multilevel questions about interference, awareness, distributed work, and control allocation | Does not validate PIANO or demonstrate clinical benefit from concentrated control |
| Robotic work system | Catchpole et al. [2, 6]; clinical observation and human-factors analysis | Robotics changes workload, communication, room configuration, disruptions, and learning | Supports treating robotics as a sociotechnical reallocation of work | Does not imply a uniform direction of effect across phases, teams, or procedures |
| Work-system interventions | Kanji et al. [14]; systematic review | Challenges were described more often than team training, checklist, or workspace interventions were evaluated | Supports intervention testing at system level | Does not establish which intervention is effective in a specific setting |
| Surgeon-controlled visualisation | Jaspers et al. [11]; review of camera and instrument holders | Stable surgeon-directed views may shorten the command loop; usability and integration determine value | Supports direct control of a selected function as a testable configuration | Does not establish whole-procedure independence or patient benefit |
| Four-arm "solo" feasibility | Park et al. [12]; prospective selected-case lobectomy study | One console surgeon controlled camera and operative arms in a defined thoracic workflow | Supports technical feasibility of concentrated control in selected cases | Does not remove staffed perioperative or rescue dependence and does not prove comparative superiority |
| Robotic camera manipulator | Takahashi et al. [13]; comparative selected hernia cases | Operations were completed without a conventional camera assistant, with setup and control costs | Supports internalisation of camera control in a bounded workflow | Generalisability, emergency resilience, and system-level benefit remain uncertain |
| Musician motor adaptation | Munte et al. [26], Furuya et al. [27]; neuroscience review and empirical motor study | Long practice is associated with neural adaptation and individuated finger control | Supports the pedagogical proposition that component actions can become integrated through practice | Does not show that pianists have innate or transferable robotic-surgery competence |
| Surgical workload | Law et al. [28] and Catchpole et al. [6]; workload and observational studies | Workload varies by approach, phase, experience, and team configuration | Supports phase- and role-specific workload measurement | Does not establish one universal workload advantage of robotics |
| Haptic feedback | Okamura [15], van der Meijden and Schijven [16], Amirabdollahian et al. [17]; reviews | Direct haptic feedback is often limited; effects vary by task, modality, fidelity, and experience | Supports evaluating the complete perception-action loop | Does not show that adding any haptic channel necessarily improves clinical performance |
| Training architecture | Schreuder et al. [43], Ahmed et al. [44], Del Calvo et al. [45]; review and consensus literature | Structured curricula, simulation, proficiency progression, and graduated responsibility are recurrent recommendations | Supports layered training beyond case counts | Simulation completion does not establish transfer to unsupervised clinical competence |
| GEARS | Goh et al. [48]; initial clinical validation study | Scores differentiated experience groups with internal-consistency and inter-rater evidence | Supports global technical formative assessment within sampled settings | Limited procedure detail; no direct assessment of teamwork, entrustment, or patient outcome |
| Assessment landscape | Kutana et al. [53], Boal et al. [54], Laughlin et al. [55]; reviews | Manual, automated, and AI tools are heterogeneous and transfer is uncertain | Supports complementary, intended-use validation rather than tool hierarchy | No reviewed tool alone supports universal accreditation or competence claims |
| R-OSATS | Siddiqui et al. [49]; multi-institutional, 105 participants, five dry-lab drills | Known-groups differences and good inter- and intra-rater evidence were reported | Supports standardised dry-lab task assessment | Does not establish whole-procedure or workplace competence |
| ARCS | Liu et al. [50]; 15 participants, three standardised tasks, three blinded raters | Several console domains differentiated experience groups; rater concordance was reported | Supports diagnostic feedback on distinct console mechanics | Small model-task sample limits clinical and cross-procedure generalisation |
| RO-SCORE | Gerull et al. [51]; pre/post cohort of 31 robotic-naive residents | Operative ratings and workload improved after simulator curriculum | Supports feasibility of pairing simulator training with workplace entrustment assessment | No concurrent control group; change cannot be attributed causally to the curriculum alone |
| RAS-NOTECHS | Schreyer et al. [29]; preliminary validation study | Multidisciplinary non-technical performance could be observed in robotic settings | Supports a separate team-level assessment construct | Does not substitute for console technical or procedure-specific assessment |

| Topic | Source, design, and unit of observation | Principal observation | Supported inference | Explicit boundary |
| --- | --- | --- | --- | --- |
| Initial CARS | Elzein et al. [57]; single centre, 29 residents, in-person assessment and 20 blinded operative videos | CARS differentiated beginner, intermediate, advanced resident, and attending performance in sampled settings; inter-rater reliability was moderate in analysed hernia videos | Provides initial validation evidence for competency-based, domain-specific assessment of resident robotic performer competence | Broader procedures, repeated cases, calibrated raters, and external settings remain necessary before stand-alone high-stakes use |
| CARS 2.0 | Goyal et al. [58]; global cross-sectional blinded video study, 320 respondents, one edited multi-operator case | Scores varied significantly across all seven video-visible competencies according to evaluator experience; the rubric was feasible for global blinded video review | Extends response-process, deployment, and generalisability evidence supporting CARS as a competency-oriented framework for robotic technical performance | One edited case does not by itself establish cross-case reliability, predictive validity, or a stand-alone credentialing threshold |
| Automated feedback | Brown and Kuchenbecker [61]; controlled novice study, 29 participants | Automated GEARS-domain feedback changed aspects of self-appraisal but did not accelerate early psychomotor learning | Supports using automated scores as an adjunct to interpretable coaching | A consistent score is not equivalent to effective instruction or clinical transfer |
| Dual-console teaching | Smith et al. [63], Cristofari et al. [64]; teaching study and qualitative video analysis | Teaching depended on communication, control transfer, and negotiated trainee participation | Supports direct observation and graduated supervision through dual-console work | A second console does not itself establish instructional quality or unambiguous responsibility |
| Team interaction | Gillespie et al. [30], Nyein et al. [31]; mixed-studies and scoping reviews | Evidence clusters around communication, roles, shared models, flow disruption, and education; definitions remain inconsistent | Supports measuring collective performance separately from console skill | No single teamwork variable or tool captures the full robotic work system |
| Bedside expertise | Britton et al. [32], Brian et al. [33], Garbens et al. [34]; critical synthesis, Delphi, and clinical cohort | Bedside work includes technical, anticipatory, and non-technical expertise; experience may affect early console performance | Supports explicit sampling of bedside competence and workload | Concentrated console control does not make bedside rescue expertise dispensable |
| Bounded autonomy | Yang et al. [18], Haidegger [19], Lee et al. [20], Shademan et al. [21], Saeidi et al. [22]; frameworks, review, and preclinical studies | Autonomy is function- and level-specific; most cleared systems were low autonomy; defined subtasks were achieved under controlled supervision | Supports declared operating domains, supervision, handover, and bounded task claims | Does not establish autonomous management of an operation or patient |
| Data-rich surgery and governance | Maier-Hein et al. [23], Hashimoto et al. [24], Marcus et al. [25]; perspective and governance framework | Data science may support recognition and decision support; staged robotics evaluation requires transparency and monitoring | Supports prospective, staged, use-specific evaluation | Product descriptions or retrospective accuracy do not establish clinical utility, fairness, or safe deployment |
| Bedside-assistant staffing | Gosavi et al. [65]; systematic review, three comparative observational studies | Possible time-based efficiency gains with dedicated or non-physician models; safety reporting was heterogeneous | Supports explicit evaluation of bedside staffing models | Confounding, few studies, and heterogeneous outcomes preclude claims of equivalence or safety benefit |
| Technology-enhanced basic training | Shakir et al. [46]; systematic review, 17 studies, 484 participants | Benefits were concentrated at learning outcomes; no level 3 or 4 outcomes were reported | Supports structured technology-enhanced practice and objective feedback | Does not establish workplace transfer, independent competence, or patient benefit |
| VR skill transfer | Alsamhori and Khadra [47]; systematic review and meta-analysis, seven pooled trials | Global skill g 0.77 (95% CI -0.64 to 2.18) and time g 0.47 (95% CI -0.35 to 1.29) were imprecise and non-significant | Suggests possible transfer but substantial uncertainty | Does not support a definitive transfer effect or credentialing inference |
| Supervised surgeon workload | Ishiyama et al. [42]; single-centre study, 72 cases and 10 surgeons | Junior surgeons reported higher workload in several domains under direct supervision | Supports role-, phase-, and experience-specific workload measurement | Comparable short-term outcomes under supervision do not establish independent safety or causal effects |
| Setup and docking assessment | Shakir et al. [52]; pre/post validity study, 51 novices | Inter-rater ICC 0.91 (95% CI 0.75-0.97) across 12 videos; scores increased after training | Provides preliminary validity evidence for structured platform-specific assessment | Single-course, one-platform evidence is insufficient for accreditation or cross-platform use |
| Explainable kinematic assessment | Xuan et al. [62]; JIGSAWS benchmark, 103 trials from 40 subjects | Pooled subject-independent accuracy was 97.1%; off-task transfer fell to 61-95% | Supports explainable research benchmarks for kinematic feedback | Dry-lab benchmark performance does not establish external clinical validity or decision consequences |
| Emergency undocking training | Xiao et al. [66]; prospective simulation study, 30 participants and 12 cycles | Confidence, knowledge, time, and simulated blood-loss estimates improved after interprofessional training | Supports feasibility of team-based rescue rehearsal | Small simulation study without patient outcomes or an untreated comparator |
| Updated haptic evidence | Abayazid [40]; systematic review across medical robotics | Benefits remained task-dependent and clinical translation was constrained by sensing, control, human-factors, and regulatory limitations | Supports information-oriented evaluation of haptic feedback | Cross-domain review does not establish benefit for a specific surgical platform or procedure |

Table S5. Domain derivation, rejected alternatives, and planned observables

| Extracted problem | Conceptual anchor and representative evidence | Domain decision | Alternative or overlap rejected | Negative case used to test the boundary | Candidate observable data |
| --- | --- | --- | --- | --- | --- |
| Control can be concentrated for one task but not another | Phase-varying workflow, surgeon-controlled visualisation, selected four-arm and camera-manipulator feasibility [2, 6, 11–14] | Retain Phase-specific control at task/phase level | Rejected whole-operation labels such as "solo surgery" and arm count as sufficient definitions | Stable dissection benefits from direct camera/retraction, while bleeding or stapling requires rapid redistribution | Control-state logs, request-to-action latency, camera/exposure stability, conflicts, idle time, recovery time |
| Command quality depends on what the interface transmits and returns | Multiple-resource theory, situation awareness, haptic reviews, motion scaling [7, 8, 15–17] | Retain Interface fidelity at human-machine loop level | Rejected degrees of freedom, feature count, or visual resolution as standalone proxies for control quality | Added functions increase error when mode state, force, uncertainty, or failure is hidden | Input-output mapping, mode state, haptic/visual cues, clutch burden, mode errors, detection and recovery latency |
| Authority and action can move among console, bedside, software, and automation | Types and levels of automation, surgical autonomy frameworks, IDEAL robotics [10, 18–25] | Retain Agency allocation at authority/governance level | Rejected a single surgeon-versus-robot autonomy axis | An accurate automated or console function remains unsafe when initiation, veto, override, handover, or rescue is ambiguous | Control transfers, permissions, automation state, override use, handover time, escalation, rescue ownership |
| Fluent coordination can coexist with overload or brittle automaticity | Multiple-resource theory, musician motor evidence, surgical workload studies [7, 26–28] | Retain Neuromotor integration at operator level | Rejected speed, smoothness, or bimanual economy as sufficient indicators of integrated competence | Fast completion masks tunnelling, excessive force, mode error, or poor recovery under perturbation | Bimanual and pedal coordination; gaze; workload; instrument kinematics and visually observed tissue deformation as indirect, non-calibrated loading proxies; mode awareness; and error detection and recovery. |
| Local control is nested in distributed cognition and team rescue | Distributed cognition, robotic team studies, RAS-NOTECHS, bedside-assistant literature [2, 9, 29–34] | Retain Orchestrated teamwork at team/system level | Rejected generic "teamwork" detached from phase, agency, and rescue | Console latency improves while bedside workload, shared awareness, or emergency response worsens | Closed-loop communication, shared plan, team workload, non-technical skills, flow disruption, fault and conversion response |

1. A domain had to answer one of the three review questions.
2. It had to occupy a distinguishable analytical level and add information not already captured by another domain.
3. It had to be expressible through observable or recordable indicators.
4. It had to generate at least one proposition capable of being contradicted.
5. It had to survive a negative case in which more concentrated control was neutral or harmful.
6. Domain labels were assigned only after conceptual grouping. No concept was retained merely to complete the acronym PIANO.
